# A Step Toward the Exploration of Better Spirometric Parameters for Early Diagnosis of Pulmonary Dysfunction in Persons With Type 2 Diabetes Mellitus

**DOI:** 10.7759/cureus.26622

**Published:** 2022-07-06

**Authors:** Rashmi R Dash, Bandita Panda, Madhuri Panigrahi, Biswaranjan Nayak

**Affiliations:** 1 Metabolic Disorders and Neonatal Physiology, Kalinga Institute of Medical Sciences, Bhubaneswar, IND; 2 Research and Development, Kalinga Institute of Medical Sciences, Bhubaneswar, IND; 3 Physiology, Kalinga Institute of Medical Sciences, Bhubaneswar, IND; 4 Neurosurgery, AMRI (Advanced Medical Research Institute) Hospitals, Bhubaneswar, IND

**Keywords:** type 2 diabetes mellites, type 2 diabetes, body mass index (bmi), forced expiratory volume (fev1), slow vital capacity (svc), forced vital capacity (fvc)

## Abstract

Objectives: This study aims to determine the forced vital capacity (FVC) and slow vital capacity (SVC) along with other pulmonary functions in Indian diabetic patients for early diagnosis of pulmonary function reduction and to compare the ratios of forced expiratory volume in one second (FEV1) with FVC and SVC (FEV1/FVC and FEV1/SVC) in diabetic patients.

Materials and methods: A prospective observational study was carried out in the physiology department for two years after the approval of the institutional ethics committee. The study included 90 type 2 diabetes mellitus patients previously diagnosed by the physician and 90 age and sex-matched controls for spirometric tests. Medspiror having Helios 401 software (Recorders & Medicare Systems Pvt. Ltd., Panchkula, India) was used to assess the pulmonary function in all subjects. A comparison of dynamic pulmonary function parameters among non-diabetic and diabetic groups and non-obese vs. overweight/obese individuals of the diabetic group has been done. FEV1/FVC ratio vs. FEV1/SVC ratio comparison was conducted between the non-obese vs. the overweight/obese group in diabetic patients.

Results: A significant variation in FEV1 and FVC was observed in the type 2 diabetic group as compared to the non-diabetic group. However, in the case of type 2 diabetic subjects, FEV1/FVC ratio was almost constant in both BMI groups, whereas the FEV1/SVC ratio increased in the overweight/obese group.

Conclusion: Type 2 diabetes mellitus accounts for a predictive factor for worsening pulmonary function. SVC, particularly the FEV1/SVC ratio, can be an earlier diagnostic marker for pulmonary dysfunction in diabetic subjects as this ratio changes even with a constant FEV1/FVC ratio.

## Introduction

Diabetes affects about 422 million people worldwide and it mostly affects low and middle-income countries. Each year, 1.5 million deaths are directly attributed to diabetes [1]. The prevalence of diabetes has been steadily increasing over the past few decades. Prolonged elevated levels of blood glucose lead to serious damage to the heart, blood vessels, eyes, kidneys, and nerves and enhance the risk of long-term complications such as neurological and vascular complications. Microvascular complications lead to kidney failure and blindness and macrovascular complications lead to coronary heart disease, cerebrovascular disease, and peripheral artery disease [2,3]. Lung disorders associated with diabetes have recently been challenged by newer studies of disease development during the coronavirus disease 2019 (COVID-19) pandemic. The progression of pulmonary diseases and pulmonary fibrosis in association with diabetes represents a higher mortality rate. The study of pulmonary function parameters (both forced and slow vital capacity and ratios with forced expiratory volume) is the main focus area to address these complications and reduce or avoid these health-threatening problems [4,5]. However, the cause of the reduction in pulmonary function remains unclear. A possible association between obesity, metabolic syndrome, and pulmonary impairment is recognized in a restrictive pattern [6]. Vital capacity (VC) is considered as the volume of air that flows between a maximal inspiratory maneuver and a maximal expiratory maneuver. Forced vital capacity (FVC) and slow vital capacity (SVC) maneuvers are the parameters to determine VC. Obese and diabetic individuals have reduced pulmonary function parameters like FVC, forced expiratory volume in one second (FEV1), predicted FVC%, and predicted FEV1% [7]. SVC parameters have not been explored properly in diabetic obese patients. The present study aimed to determine FVC and SVC along with other pulmonary functions in diabetic patients of the Indian population for early diagnosis of pulmonary function reduction.

## Materials and methods

A prospective study was conducted on pulmonary function tests of diabetic patients at the Physiology Department of SCB Medical College, Cuttack after Institutional Ethics Committee approval (IEC/IRB No.: 11/09-10-2013). Patients with type 2 diabetes between the age group of 30 and 60 years, without any complications, who consented to participate, were included in the study. For exclusion criteria, subjects having cardio-respiratory diseases, respiratory allergy, history of smoking and alcoholism, and acute respiratory infection were excluded. The sample comprised 180 individuals, out of which 90 were type 2 diabetic patients (69 males and 21 females) and 90 were healthy controls (69 males and 21 females). The diabetic group was categorized in response to body mass index: ≥25 kg/m^2^ (obese) and <25 kg/m^2^ (non-obese). The pulmonary function was assessed in all subjects by spirometry (slow and forced maneuvers in Medspiror having Helios 401 software (Recorders & Medicare Systems Pvt. Ltd., Panchkula, India)) with measurements of specific airway resistance and alveolar volume from single breath lung diffusing capacity, which measures all lung volumes. Spirometric recordings were taken three hours after breakfast. The glycemic status of a diabetic patient was determined by fasting blood glucose (FBG) after 12 hours of fast and postprandial blood glucose (PPBG) by glucose oxidase and peroxidase method as conducted after two hours of the meal. Fasting blood sugar (FBS) value of more than 126 mg/dL was considered a diagnostic value of diabetes and a PPBG value of more than 200 mg/dl was considered a diagnostic value of diabetes. With the consent of patients, the highest reading of three trials in a sitting posture at room temperature three hours after breakfast was recorded.

In the first step, maximum inhalation followed by maximum exhalation was conducted through the mouthpiece for the measurement of FVC. In the second step, SVC was measured in normal breathing for three to four times followed by maximum inhalation and again normal breathing for some time. To measure maximum voluntary ventilation (MVV), the patient was asked to take deep inspiration followed by forceful expiration as quickly as possible for 15 seconds.

The following parameters were measured by using Medspiror Helios 401 software: FVC (ml), FEV1 (ml), percentage of FEV1 (FEV1% = FEV1 x 100), maximum mid-expiratory flow rate (MMEFR; forced expiratory flow (FEF) rate 25-75%), peak expiratory flow rate (PEFR; L/min), SVC, and MVV.

Statistical analysis

All the variable data were analyzed using statistical analysis tools such as mean, standard deviation, p-value (p-value < 0.05 was considered to be significant), and correlation analysis for data validation. All statistical analyses were done using standard statistical software STATA version 13.1 (StataCorp LLC, College Station, TX).

## Results

The study included 180 subjects for a spirometry test, of which 90 were from the diabetic group and 90 were from the non-diabetic group. Anthropometric parameters like age, height, weight, and BMI were recorded in both diabetic and control groups. In both groups, the average age varied between 46 and 49 years with an average weight of 61 kg and an average BMI of 24 kg/m^2^ ± 3. Table 1 illustrates the anthropometric parameters of the diabetic and control groups showing no significant differences in age, height, weight, and BMI between the diabetic and control groups.

**Table 1:** Comparison of anthropometric parameters between the diabetic and non-diabetic groups. Data are presented as mean ± SD. P > 0.05: not significant (NS); P < 0.05: significant (S); P < 0.01: highly significant (HS)*.

Parameters	Diabetes group (n = 90), mean and SD	Non-diabetic group (n = 90), mean and SD	P-value (p < 0.05), significant
Age (years)	48.34 ± 8.24	46.42 ± 9.06	0.138 (NS)
Height (m)	1.59 ± 0.08	1.6 ± 0.06	0.785 (NS)
Weight (kg)	61.27 ± 9.68	62.03 ± 10.58	0.613 (NS)
BMI (kg/m^2^)	24.13 ± 3.24	24.28 ± 3.63	0.558 (NS)

Analysis of pulmonary functions in diabetic and control groups

The mean value of pulmonary function parameters like SVC (L) and expiratory reserve volume (ERV) (L) of both groups is presented in Table 2. Parameters like SVC and ERV are found to be lower in the diabetic group in comparison to the control group. In the case of the diabetic group, SVC values were significantly lower than the control group.

**Table 2:** Comparison of pulmonary function test static parameters between the diabetic and non-diabetic groups. Data are presented as mean ± SD. P > 0.05: not significant (NS); P < 0.05: significant (S); P < 0.01: highly significant (HS)*. SVC: slow vital capacity; ERV: expiratory reserve volume.

Parameters	Diabetes group (n = 90), mean ± SD	Non-diabetic group (n = 90), mean ± SD	P-value
SVC (L)	2.60 ± 0.67	2.94 ± 0.63	0.001 (HS)*
ERV (L)	0.59 ± 0.56	0.74 ± 0.54	0.077 (NS)

All the parameters of pulmonary function such as FEV1, FVC, FEF rate over the middle 50% of the FVC, PEFR, and MVV are shown in Table 3. All parameters except FEV1/FVC were statistically significantly lower in the diabetic group as compared to the control group.

**Table 3:** Comparison of pulmonary function test dynamic parameters between diabetic and non-diabetic groups. FEV1: forced expiratory volume in one second; FVC: forced vital capacity; FEF_25-75%_: forced expiratory flow rate over the middle 50% of the FVC; PEFR: peak expiratory flow rate; MVV: maximum voluntary ventilation; NS: not significant; HS: highly significant.

Parameters	Diabetes group (n = 90), mean ± SD	Non-diabetic group (n = 90), mean ± SD	P-value
FEV1	1.91 ± 0.54	2.45 ± 0.48	0.000 (HS)*
FVC	2.17 ± 0.59	2.73 ±.49	0.000 (HS)*
FEV1/FVC (%)	88.05 ± 9.26	89.82 ± 6.97	0.149 (NS)
FEF_25-75% _(L/sec)	2.37 ± 1.03	3.16 ± 1.08	0.000 (HS)*
PEFR (L/sec)	4.35 ± 1.56	5.42 ± 1.66	0.000 (HS)*
MVV (L/min)	79.93 ± 23.67	92.71 ± 23.32	0.000 (HS)*

Correlation of lung function parameters with glycemic parameters

FBS correlated positively with FEV1, FEV1/FVC%, PEFR, MVV, ERV, inspiratory reserve volume (IRV), and FEV1/FVC% but negatively with FVC, SVC, and inspiratory capacity (IC). There was a negative correlation between FEV1/SVC% and post-prandial blood sugar (PPBS) (Table 4).

**Table 4:** Correlation between FBG, PPBG, and pulmonary function test parameters. ** Correlation is significant at the 0.01 level (2-tailed). * Correlation is significant at the 0.05 level (2-tailed). FBS: fasting blood sugar; PPBG: postprandial blood glucose; FEV1: forced expiratory volume in one second; FVC: forced vital capacity; FEF: forced expiratory flow rate; PEFR: peak expiratory flow rate; MVV: maximum voluntary ventilation; SVC: slow vital capacity; ERV: expiratory reserve volume; IRV: inspiratory reserve volume; IC: inspiratory capacity.

Pulmonary function test parameters		FBS (mg/dl)	PPBS (mg/dl)
FEV1 (L/sec)	r	0.098	0.044
	P-value	0.360	0.679
FVC (L)	r	-0.008	0.004
	P-value	0.941	0.971
FEV1/FVC%	r	0.097	0.080
	P-value	0.365	0.451
FEF (25-75%)	r	0.104	0.138
	P-value	0.330	0.195
PEFR (L/sec)	r	0.214^*^	0.102
	P-value	0.043	0.338
MVV (L/min)	r	0.153	0.310^**^
	P-value	0.149	0.003
SVC (L)	r	-0.014	0.256^*^
	P-value	0.894	0.015
ERV (L)	r	0.169	0.080
	P-value	0.112	0.455
IRV (L)	r	0.047	0.274^**^
	P-value	0.657	0.009
IC (L)	r	-0.117	0.162
	P-value	0.270	0.127
FEV1/SVC%	r	0.111	-0.153
	P-value	0.299	0.150

The respiratory impairment in the diabetic group was observed to be restrictive in 55.5% of the population, and in 30% of cases, it was normal, while 5% of cases had an obstructive impairment and 8% of cases had both obstructive and restrictive impairment (Table 5).

**Table 5:** Types of respiratory impairment in the diabetic group.

Types of respiratory impairment	No. of cases	Percentage of cases
Normal	27	30%
Restrictive impairment	50	55.56%
Obstructive impairment	5	5.55%
Obstructive and restrictive impairment	8	8.89%
Total	90	100%

Comparative pulmonary function ​parameters in two BMI groups of diabetic population

**Figure 1:**
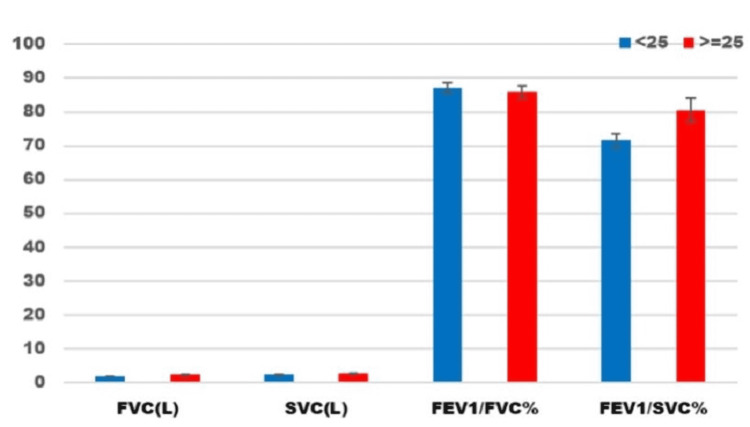
Comparative pulmonary function parameters among non-obese (<25 kg/m2) and obese (>25 kg/m2) patients in the diabetic group. FVC: forced vital capacity; SVC: slow vital capacity; FEV1: forced expiratory volume in one second.

A comparison of pulmonary function parameters in the obese/overweight (≥25 kg/m^2^) diabetic group and the normal diabetic group (<25 kg/m^2^) is shown in Table 6. Among the pulmonary function test parameters, FVC and SVC individually did not show any significant variation between the obese and non-obese diabetic patients. FEV1/FVC ratio was not significant, whereas the ratio of FEV1 and SVC showed a significant difference between the two groups with a p-value of 0.03. The ratio of FEV1 with SVC was higher in the obese diabetic group (>25 kg/m^2^) in comparison to the ratio of FEV1 with FVC (Table 6 and Figure 1).

**Table 6:** Comparative pulmonary function parameters among non-obese (<25 kg/m2) vs. overweight/obese (>25 kg/m2) patients in the diabetic group. FVC: forced vital capacity; FEV1: forced expiratory volume in one second; SVC: slow vital capacity; NS: not significant; HS: highly significant.

Parameters	BMI group (kg/m^2^)	Mean ± SD	P-value (2-tailed)
FVC (L)	<25 (n = 54)	2.01 ± 0.53	0.000
	≥25 (n = 36)	2.44 ± 0.58	
FEV1/FVC	<25 (n = 54)	87.10 ± 11.41	0.628 (NS)
	≥25 (n = 36)	85.92 ± 11.11	
SVC (L)	<25 (n = 54)	2.46 ± 0.62	0.092 (NS)
	≥25 (n = 36)	2.70 ± 0.70	
FEV1/SVC	<25 (n = 54)	71.65 ± 14.55	0.031 (HS)
	≥25 (n = 36)	80.49 ± 21.17	

## Discussion

The present study was done to observe the pulmonary impairment in type 2 diabetic patients aged 30-60 years for risk assessment of pulmonary function. A total of 90 diabetic patients were included in the study, and 90 healthy subjects were included as controls for comparison. The anthropometric, biochemical, and pulmonary function parameters were recorded to determine the pulmonary diagnostic criteria for diabetic patients based on their physiological and anthropometric parameters.

The anthropometric parameters between the diabetic and control groups did not show any significant differences in age, height, weight, and BMI in the present study groups. Among the pulmonary static parameters, such as SVC and ERV, SVC showed significant variation between the diabetic and control groups. A significant reduction in SVC value was observed in the diabetic group compared to controls, whereas the decline in ERV value was not significant. Devulapally et al. reported that anthropometric parameters such as weight, skinfold thickness of the abdomen, forearm, and circumference of the midarm and forearm are significantly higher in the diabetic group in comparison to the control group. BMI, body fat, and muscle volume showed significantly higher variation in the diabetic group as compared to the control group [8].

The effect of VC of diabetic patients on pulmonary function has been reported by many authors [6,7]. The VC decreases with increasing BMI. It has been reported that obesity reduces lung compliance and it also decreases VC. Chest wall compliance varies in different studies [9-11]. Moreover, it affects airway closure, pulmonary gas trapping, diffuse microatelectasis, and relatively intrathoracic blood volume increase and enhances the respiratory system elastance [12,13].

Our study shows a significant correlation between FBS and PEFR. There was a positive correlation between PPBG and MVV, SVC, and IRV. Our findings are similar to the results of Davis et al., in which lung function consistently declined as predicted by poor glycemic control and duration of diabetes [14].

In the present study, a negative correlation was observed between PPBS and FEV1/SVC%, whereas there was no significant correlation between blood sugar and FEV1/FVC%. Lung function declines in the case of diabetic patients because of the rich vascularity and abundance of connective tissues (collagen and elastin). Microvascular complications and proliferation of extracellular connective tissues in the lungs lead to a decline in lung function in a restrictive pattern. Interstitial lung disease (ILD) includes different degrees of inflammation and fibrosis in the pulmonary parenchyma. The mortality rate is higher in idiopathic pulmonary fibrosis (IPF), which is one of the common types of idiopathic interstitial pneumonia, and chronic progressive fibrosis leads to progressive respiratory failure [15].

Studies have proposed two major mechanisms by which diabetes leads to lung diseases. Thorax and lungs are rich in collagen and elastin and the non-enzymatic glycation of these compounds could result in stiffening of the thoracic cage and lung parenchymal tissue resulting in restrictive physiology. Microvascular damage and thickening of alveolar epithelium in the lungs are the cause of declined lung function leading to nephropathy, retinopathy, and neuropathy in diabetic patients. The combination of thickening alveolar wall and reduced perfusion results in ventilation-perfusion mismatch resulting in impaired diffusion capacity [16].

In the case of diabetic patients associated with a ventilatory defect on the pulmonary function, lung function showed a restrictive pattern of ventilatory defects. A declined FEV1 was observed in patients with diabetes at a rate of two to three times faster than that of normal, non-diabetic, non-smoking subjects [17]. On average, FEV1 reduces by 25-30 mL/year in non-smoking, healthy adults, and around 71 mL/year decline is seen in FEV1 subjects with diabetes mellitus [16]. In patients with diabetes mellitus, a decline in FVC and dynamic lung compliance, which is probably related to peripheral airway obstruction, has been reported [16]. Shah et al. reported reduced FVC and FEV1 significantly in patients (40-60 years) with type 2 diabetes mellitus as compared to healthy controls, except FEV1/FVC ratio, which was similar in both groups [17].

Ventilatory dysfunction in diabetic subjects is predominantly restrictive, as shown by the preserved ratio of FEV1/FVC%. Our results are similar to the findings of large population studies in Australia, Denmark, and the United States, including those in which measured values of FEV1/FVC% were compared with predicted values [12,18,19].

In the present study, BMI had a positive correlation with FVC, SVC, and FEV1/SVC, and no significant relation was observed between BMI and FEV1/FVC. Hence, in the case of diabetic patients with high BMI, FEV1/SVC had a greater diagnostic value than FEV1/FVC. Similar results were observed in a population-based study in Southern Australia, where BMI, coronary artery disease, and age were found to be significant determinants of ventilatory function [7]. The effect of BMI on reducing lung function has been well documented [20-24]. Chest wall compliance and increased airway resistance are the major factors responsible for this reduced pulmonary function. The effect of BMI on lung function is associated with metabolic syndrome in which low-grade inflammation plays a major role in the development of diabetes as well as reduced lung function [24].

The limitation of the study was the inadequate sample size in the case of diabetic patients with high BMI with an equal number of males and females.

## Conclusions

Pulmonary function in terms of both static and dynamic parameters is demonstrated to be reduced and restrictive in the diabetic group as compared to the healthy controls. Type 2 diabetes mellitus accounts as a predictive factor for worsening pulmonary function. In the diabetic group, BMI added obstruction in SVC and FVC parameters. Among the ratio of FEV1 with FVC and SVC, the ratio of FEV1 with SVC (FEV/SVC) gives significant differentiation in the obese diabetic group than the non-obese diabetic group. Thus, the SVC and the FEV1/SVC ratio can be considered early diagnostic indicators for pulmonary dysfunction in obese diabetic patients.
